# Eosinophilic COPD: a Separate Disease Phenotype Warranting Specific Treatment

**Published:** 2017

**Authors:** Leo Koenderman

**Affiliations:** Department of Respiratory Medicine, University Medical Center Utrecht, The Netherlands.

For a long time disease severity of COPD was characterized by the GOLD criteria. These criteria are based on lung function criteria and do not take differences in inflammatory response into account. It is generally agreed upon that COPD is characterized by different pathogenetic mechanisms either resulting in a bronchitis type of COPD or in a emphysematous type. This difference is important as it is to be expected that the bronchitis type will benefit more from anti-inflammatory therapy.

Recently, it has become clear that the bronchitis type of COPD can divided into a phenotype with a clear eosinophilic signal and a phenotype with pure neutrophil signature. It is unclear what the underlying mechanisms are, but the eosinophilic type might benefit from therapies directed against eosinophils such as treatment with monoclonal antibodies directed against IL-5 (e.g. mepolizumab).

The presentation will review the latest data on the putative success of application of eosinophil targeted therapy in eosinophilic COPD.

